# Supplementary material for: Mortality in Hemodialysis Patients with COVID-19, the Effect of Paricalcitol or Calcimimetics
Source: Nutrients. 2021 Jul 26;13(8):2559. doi: 10.3390/nu13082559 (PMC8401800; doi:10.3390/nu13082559)
Supplement: Supplementary file 1 [file nutrients-13-02559-s001.zip › nutrients-1288459-supplementary.pdf]

Supplementary Material

Table S1 . Univariant logistic regression analyses of hospital admission and care requirements according to serum VD levels in COVID-19 positive HD patients

		**OR [CI 95%]**	***p***
**Hospital admission**	25-OH serum levels (ng/ml; *Ref. <20)*	-	-
	20-30	0.595 [0.289-1.226]	0.159
	>30	0.992 [0.446-2.207]	0.984
**ICU admission**			
	25-OH serum levels (ng/ml; *Ref. <20)*	-	-
	20-30	0.783 [0.157-3.905]	0.765
	>30	0.878 [0.175-4.395]	0.874
**Non-invasive ventilation requirements**			
	25-OH serum levels (ng/ml; *Ref. <20)*	-	-
	20-30	1.150 [0.536-2.467]	0.719
	>30	1.858 [0.892-3.874]	0.098
**Mechanical ventilation requirement**			
	25-OH serum levels (ng/ml; *Ref. <20)*		
	20-30	0.447 [0.052-3.810]	0,461
	>30	0.525 [0.061-4.492]	0,556

Table S2. Baseline characteristics of patients treated with paricalcitol. Death 81 patients. *N* = 285 .

	**Paricalcitol** *N* **= 94** **Death: 16** **Mortality : 17%**	**No paricalcitol** *N* **= 191** **Death= 65** **Mortality=34%**	***p***
Age (years, mean ± SD)	72.3 ± 13.1	72.4 ± 12.4	0.953
Female gender	31 (33%)	53 (27.3%)	0.322
Body mass index (kg/m^2, mean ± SD)	26.8 ± 5.29	25.04 ± 5.47	0.012
Dialysis vintage (years, median [p25-75])	2.5 [3.6 – 6.3]	2.5 [3.3 - 4.7]	0.178
Native fistulae as vascular access (*N* = 261)	55/94 (58.5%)	99/167 (59.3%)	0.903
Current Smoking status (*N* = 208)	31/64 (48.4%)	71/144 (49.3%)	0.908
Alcohol (*N* = 177)	1/61 (1.6%)	16/116 (13.8%)	0.009
Previous transplant (*N* = 261)	10/94 (10.6%)	10 /167(6.0%)	0.175
Diabetes Mellitus	42/94 (44.7%)	94/191 (49.2%)	0.471
Arterial hypertension	33/94(35.1%)	93/191 (48.7%)	0.030
Tuberculosis (*N* = 261)	1/94 (1.1%)	4/167 (2.4%)	0.451
Dyslipemia	28/94 (29.8%)	77/191(40.3%)	0.083
Lung disease	20/94 (21.3%)	46/191 (24.1%)	0.597
Ischemic cardiopathy	29/94 (30.9%)	64/191 (33.5%)	0.653
Calcidiol	4/94 (4.3%)	28/191 (14.7%)	0.009
Calcimimetics	26/94 (27.7%)	23/191 (12%)	0.001
25-OH-D (ng/ml, , mean ± SD) (*N* = 252)	20.06 ± 13.7	21.1 ± 13.3	0.189
< 20 ng/ml	59/88(67%)	90/164 (54.9%)	
20-30 ng/ml	13/88(14.8%)	39/164 (23.8%)	
>30 ng/ml	16/88 (18.2%)	35/164(21.30%)	
PTH (ng/L, median [p25-75])	386 [196 - 479]	285 [105 - 343]	0.013
Calcium (mg/dl, mean ± SD)	8.8 ± 0.8	8.8 ± 0.6	0.870
Phosphate (mg/dl, mean ± SD)	4.2 ± 0.9	3.9 ± 1.2	0.125

ACEIs	24/94 (25.5%)	37/191 (19.4%)	0.233
ARBs	7/94 (7.4%)	24/191 (12.6%)	0.192
NSAIDs	8/94 (8.5%)	13/191 (6.8%)	0.605
Albumin baseline (g/dl, mean ± SD)	3.8 ± 0.4	3.7 ± 0.3	0.724
C-reactive protein (mg/L, median [p25-75]) at Hospital admission	77.2 [49.7-104.7]	41.5 [28.2-54.9]	0.010
Ageusia (*N* = 93)	1/15 (6.7%)	3/78(3.8%)	0.622
Anosmia(*N* = 93)	1/15 (6.7%)	4/78 (5.1%)	0.809
Cough (*N* = 265)	44/85 (51.8%)	83/180 (46.1%)	0.390
Fever(*N* = 265)	48/85 (56.5%)	119/180 (66.1%)	0.129
Diarrhea(*N* = 265)	4/85 (4.7%)	24/180 (13.3%)	0.033
Dyspnea(*N* = 265)	26/85 (30.6%)	59/180(32.8%)	0.722
Asymptomatic (*N* = 276)	13/91 (14.3%)	22/185 (11.9%)	0.574
Pneumonia (*N* = 254)	61/83 (73.5%)	122/171 (71.3%)	0.720
Hospital admission	74/94 (78.7%)	154/191(80.6%)	0.705
ICU admission (*N* =257)	6/81 (7.4%)	9/176 (5.1%)	0.466
Endotracheal intubation (*N* = 254)	5/81 (6.2%)	5/173(2.9%)	0.210
Resolution and discharged(*N* = 264)	64/83 (77.1%)	105/181(58%)	0.003
Death	16/94 (17.0%)	65/191 (34.0%)	0.003
Hydroxychloroquine(*N* = 238)	55/76 (72.4%)	120/162 (74.1%)	0.781
Lopinavir/ Ritonavir (*N* = 239)	25/ 76 (32.9%)	67/163(41.1%)	0.224
Azithromycin(*N* = 239)	21/76(27.6%)	72/163(44.2%)	0.015
Steroids (*N* = 284)	24/94 (25.5%)	67/190 (35.3%)	0.098
Tocilizumab(*N* = 239)	6/76(7.9%)	14/163(8.6%)	0.857
Beta-Interferon(*N* = 239)	4/76 (5.3%)	8/163 (4.9%)	0.907
Remdesevir(*N* = 239)	0/76 (0.0%)	1/163 (0.6%)	0.494

*Resolutio*n* = discharged home at the time of reporting. Expressed as percentages, mean ± SD or median [IQR p25-75]. SD: standard deviation; IQR: interquartile range; ACEI: angiotensin converting enzyme inhibitors; ARB; anti-AT1 receptor antagonists, NSAIDs: non-steroidal anti-inflammatory agents.

Table S3. Baseline characteristics of patients treated with calcimimetics. Death 81 patients. *N* = 285.

	**Calcimimetics** **N = 49** **Death= 4** **Mortality = 8.2%**	**No calcimimetics** **N = 236** **Death= 77** **Mortality = 32.6%**	*p*
Age (years, mean ± SD)	67.9 ± 15.4	73.3 ± 11.8	**0.023**
Female gender	11/49 (22.4%)	72/236 (30.5%)	0.258
Body mass index (kg/m^2, mean ± SD)	27.9 ± 6.8	25.1 ± 5.0	**<0.001**
Dialysis vintage (years, median [p25-75])	4.1 [2.3 -8.5]	2.3 [1.0-4.4]	**<0.001**
Native fistulae as vascular access(*N* = 261)	30/46 (65.2%)	124/215 (57.7%)	0.345
Current Smoking status (*N* = 205)	11 /31 (35.5%)	89/174 (51.1%)	0.108
Alcohol(*N* = 177)	3/28 (10.7%)	14/149(9.4%)	0.828
Previous transplant	6/46 (13%)	14/215 (6.5%)	0.131
Diabetes Mellitus	14 /49 (28.6%)	122/239 (51.7%)	**0.003**
Arterial hypertension	16/49 (32.7%)	110/236 (46.6%)	0.073
Tuberculosis	1/46 (2.2%)	4/215 (1.9%)	0.888
Dyslipemia	16/49 (32.7%)	89/236 (37.7%)	0.504
Lung disease	6/49 (12.2%)	60/236(25.4%)	0.047
Ischemic cardiopathy	18/49 (36.7%)	75/236(31.8%)	0.501
Calcidiol	0/49 (0%)	32/236 (13.6%)	0.006
Calcitriol	2/49 (4.1%)	19/236 (8.1%)	0.333
Paricalcitol	26/49 (53.1%)	68/236 (28.8%)	0.001
Calcitriol and/or Paricalcitol	28/49 (57.1%)	83/236(35.2%)	0.004
25-OH-D (ng/ml, mean ± SD) (*N* = 252)	19.6 ± 13.2	21.0 ± 13.5	0.514

PTH (ng/L, median [p25-75])	434 [301-685]	210 [113-333]	< 0.001
Calcium (mg/dl, mean ± SD)	8.6 ± 0.7	8.8 ± 0.7	0.070
Phosphate (mg/dl, mean ± SD)	4.3 ± 1.1	3.9 ± 1.1	0.044
Angiotensin converting enzyme inhibitors	12/49 (24.5%)	49/236(20.8%)	0.563
Anti-AT1 receptor antagonists	3/49 (6.1%)	28/236 (11.9%)	0.240
Non-steroidal anti-inflammatory agents	7/49 (14.3%)	14/236 (5.9%)	0.042
Albumin baseline (g/dl, mean ± SD)	3.92 ± 0.37	3.78 ± 0.39	0.041
Hospital admission C-reactive protein (mg/L, median [p25-75])	34 [18-51]	43 [32-53]	0.792
Ageusia (*N* = 93)	0/11 (0%)	4/82 (4.9%)	0.454
Anosmia (*N* = 93)	0/11 (0%)	5/82 (6.1%)	0.400
Cough(*N* = 265)	21/46 (45.7%)	106/219(48.4%)	0.734
Fever(*N* = 265)	24/46 (65.3%)	143/219 (52.2%)	0.094
Diarrhea(*N* = 265)	2/46 (4.3%)	26/219 (11.9%)	0.131
Dyspnea(*N* = 265)	11/46 (23.9%)	74/219 (33.8%)	0.192
Asymptomatic(*N* = 276)	7/47 (14.9%)	28/229 (12.2%)	0.617
Pneumonia(*N* = 254)	31/45 (68.9%)	152/209 (72.7%)	0.603
Hospital admission (n, %)	38/49 (77.6%)	190 / 236 (80.5%)	0.638
ICU admission (n, %)(*N* = 257)	3/43 (7%)	12/214 (5.6%)	0.727
Non-invasive mechanical ventilation (n. %) (*N* = 220)	11/32 (34.4%)	63/188 (33.5%)	0.924
Endotracheal intubation (n, %)	2/43 (4.7%)	8/211 (3.8%)	0.792
Resolution and discharged	42/46 (91.3%)	127/218 (58.3%)	<0.001
Death	4/49 (8.2%)	77/236 (32.6%)	0.001
Hydroxychloroquine (*N* = 238)	32/42 (76.2%)	143/196 (73%)	0.667
Lopinavir/ Ritonavir (*N* = 239)	12/42 (28.6%)	80/197 (40.6%)	0.145
Steroids(*N* = 284)	10/48 (20.8%)	81/236(34.3%)	0.068
Azithromycin(*N* = 239)	17/42 (40.5%)	76/197 (38.6%)	0.819
Tocilizumab(*N* = 239)	3/42 (7.1%)	17/197(8.6%)	0.752
Beta-Interferon(*N* = 239)	1/42 (2.4%)	11/197 (5.6%)	0.388
Remdesevir(*N* = 239)	0/42 (0%)	1/197 (0.5%)	0.644

*Resolutio*n* = discharged home at the time of reporting. Expressed as percentages, mean ± SD or median [IQR p25-75].SD: standard deviation; IQR: interquartile range; ACEIs: angiotensin converting enzyme inhibitors; ARBs; anti-AT1 receptor antagonists, NSAIDs: non-steroidal anti-inflammatory agents.

Table S4. Baseline characteristics of patients treated with calcimimetics or paricalcitol . Death 81 patients. *N* = 285

	**Calcimimetics or paricalcitol *N* = 117 Death= 19 Mortality = 16.2%**	**No calcimimetics or paricalcitol *N* = 168 Death= 62 Mortality = 36.9%**	***p***
Age (years, mean ± SD)	71.4 ± 13	72.9 ± 12.3	0.342
Female gender	33/117 (28.2%)	50/168 (29.8%)	0.776
Body mass index (kg/m^2, mean ± SD)	27.1 ± 6.1	24.6 ± 4.6	<0.001
Dialysis vintage (years, median [*p*25-75])	5.2 [4.0-6.3]	3.8 [3.0-4.5]	0.039
Native fistulae as vascular access	67/114 (58.8%)	87/147 (59.2%)	0.947
Current Smoking status (*N* = 205)	38/79 (48%)	62/126 (49.2%)	0.878
Alcohol (*N* = 177)	4/74 (5.4%)	13/103(12.6%)	0.108
Previous transplant (*N* = 261)	12/114 (10.5%)	8/147 (5.4%)	0.126
Diabetes Mellitus	47/117 (40.2%)	89/117 (53%)	0.033
Arterial hypertension	40/117 (34.2%)	86/168 (51.2%)	0.004
Tuberculosis (*N* = 261)	1/114 (0.9%)	4/147(2.3%)	0.281
Dyslipemia	37/117 (31.6%)	68/168 (40.5%)	0.128
Lung disease	24/117 (20.5%)	42/168(25%)	0.377
Ischemic cardiopathy	38/117 (32.5%)	55/168 (32.7%)	0.963

Calcidiol	4/117 (3.4%)	28/168 (16.7%)	<0.001
Calcitriol	6/117 (5.1%)	15/168 (8.9%)	0.227
25-OH-D (ng/ml, median [p25-75])	19.7 [17.1-22.2]	21.8 [19.3-23.8]	0.219
PTH (ng/L, median [p25-75])	436 [363-508]	231.3 [197-266]	< 0.001
Calcium (mg/dl, mean ± SD)	8.7 ± 0.8	8.8 ± 0.6	0.497
Phosphate (mg/dl, mean ± SD)	4.24± 1.18	3.9 ± 1.04	0.033
Angiotensin converting enzyme inhibitors	27/117 (23.1%)	34/168(20.2%)	0.565
Anti-AT1 receptor antagonists	8/117 (6.8%)	23/168 (13.7%)	0.068
Non-steroidal anti-inflammatory agents	12/117 (10.3%)	9/168 (5.4%)	0.119
Hospital admission Albumin (g/dl, mean ± SD)	3.8 ± 0.5	3.7± 0.51	0.043
Hospital admission C-reactive protein (mg/L, median [p25-75])	66.5 [44.8-88]	43.5 [27.9-59.1]	0.080
Ageusia (*N* = 93)	1/22 (4.5%)	3/71 (4.1%)	0.948
Anosmia(*N* = 93)	1/22 (4.5%)	4/71 (5.5%)	0.843
Cough (*N* = 265)	55/108 (50.9%)	72/157(45.9%)	0.417
Fever(*N* = 265)	60/108 (55.6%)	107/157 (68.2%)	0.027
Diarrhea(*N* = 265)	6/108 (5.6%)	22/157 (14%)	0.028
Dyspnea(*N* = 265)	34/108 (31.5%)	51/157 (32.5%)	0.864
Asymptomatic(*N* = 276)	16/114 (14%)	19/162 (11.7%)	0.571
Pneumonia(*N* = 254)	77/106 (72.6%)	106/148(71.6%)	0.858
Hospital admission (n, %)	93/117 (79.5%)	135/168(80.4%)	0.857
ICU admission (n, %) (*N* = 257)	8/103 (7.8%)	7/154 (4.5%)	0.282
Non-invasive mechanical ventilation (n. %)(*N* = 220)	31/84 (36.9%)	43/136 (31.6%)	0.420
Endotracheal intubation (n, %) (*N* = 254)	6/103 (5.8%)	4/151 (2.6%)	0.201
Resolution and discharged (*N* = 264)	83/105 (79%)	86/159 (54.1%)	<0.001
Death	19/117 (16.2%)	62/168 (36.9%)	<0.001
Hydroxychloroquine (*N* = 238)	72/97 (74.2%)	103/141 (73%)	0.840
Lopinavir/ Ritonavir(*N* = 239)	31/97 (32%)	61/142 (43 %)	0.086
Steroids(*N* = 284)	29/116 (25%)	62/168 (36.9%)	0.035
Azithromycin(*N* = 239)	31/97 (32%)	62/142 (43.7%)	0.068
Tocilizumab(*N* = 239)	8/97 (8.2%)	12/142(8.5%)	0.956
Beta-Interferon(*N* = 239)	4/97 (4.1%)	8/142 (5.6%)	0.600
Remdesevir(*N* = 239)	0/97 (0%)	1/142 (0.7%)	0.408

*Resolutio*n* = discharged home at the time of reporting. Expressed as percentages, mean ± SD or median [IQR p25-75].SD: standard deviation; IQR: interquartile range; ACEIs: angiotensin converting enzyme inhibitors; ARBs; anti-AT1 receptor antagonists, NSAIDs: non-steroidal anti-inflammatory agents.

Table S5. Baseline characteristics of patients treated with paricalcitol and calcimimetics. Death 81 patients. $N = 285$ X^2 8.4, $p = 0.004$.

	**Calcimimetics and paricalcitol** ***N* = 26** **Death= 1** **Mortality = 3.8%**	**others** ***N* = 259** **Death= 80** **Mortality = 30.9%**	***p***
Age (years, mean ± SD)	67.6 ± 17	72.8 ± 11.9	0.046
Female gender	9/26 (34.6%)	74/259 (28.6%)	0.518
Body mass index (kg/m^2, mean ± SD)	27.4 ± 4.6	25.4 ± 5.5	0.079
Dialysis vintage (years, median [p25-75])	6.3 [3.6-9]	4.1 [3.5-4.8]	0.056
Native fistulae as vascular access (*N* = 261)	18/26 (69.2%)	136/235 (57.9%)	0.264
Current Smoking status (*N* = 205)	4/16 (25%)	96/189(50.8%)	0.047
Alcohol (*N* = 177)	0/15 (0%)	17/162(10.5%)	0.187
Previous transplant(*N* = 261)	4/26 (15.4%)	16/235 (6.8%)	0.119
Diabetes Mellitus	9/26 (34.6%)	127/259 (49%)	0.161
Arterial hypertension	9/26 (34.6%)	117/259 (45.2%)	0.301
Tuberculosis	1/26 (1.7%)	4/235(3.8%)	0.449
Dyslipemia	7/26 (26.9%)	98/259 (37.8%)	0.271
Lung disease	2/26 (7.7%)	64/259(24.7%)	0.050
Ischemic cardiopathy	9/26 (34.6%)	84/259 (32.4%)	0.821
Calcidiol	0/26 (0.0%)	32/259 (12.4%)	0.057
Calcitriol	0/26 (0.0%)	21/259 (8.1%)	0.131
25-OH-D (ng/ml, median [p25-75])	20.7 [14.8-26.7]	20.9 [19.1-22.6]	0.963
PTH (ng/L, median [p25-75])	514 [381-647]	298 [258-337]	< 0.001
Calcium (mg/dl, mean ± SD)	8.7 ± 0.8	8.8 ± 0.6	0.335
Phosphate (mg/dl, mean ± SD)	4.4± 1.18	4.0 ± 1.04	0.118
Angiotensin converting enzyme inhibitors	9/26 (34.6%)	52/259(20.1%)	0.085
Anti-AT1 receptor antagonists	2/26 (7.7%)	29/259 (11.2%)	0.584
Non-steroidal anti-inflammatory agents	3/26 (11.5%)	18/259 (6.9%)	0.393
Hospital admission Albumin (g/dl, mean ± SD)	3.33 ± 0.5	3.31± 0.51	0.957
Hospital admission C-reactive protein (mg/L, median [p25-75])	54.2 [11.4-97.0]	52.9 [39.4-66.4]	0.070
Ageusia (*N* = 93)	0/4 (0%)	4/89 (4.5%)	0.665
Anosmia (*N* = 93)	0/4 (0%)	5/89 (5.6%)	0.626
Cough (*N* = 265)	10/23 (43.5%)	117/242(48.3%)	0.655
Fever(*N* = 265)	12/23 (52.2%)	155/242 (64%)	0.260
Diarrhea(*N* = 265)	0/23 (0%)	28/242 (11.6%)	0.085
Dyspnea(*N* = 265)	3/23 (13%)	82/242 (33.9%)	0.041
Asymptomatic(*N* = 276)	4/24 (16.7%)	31/252 (12.3%)	0.539
Pneumonia(*N* = 254)	15/22 (68.2%)	168/232(72.4%)	0.672
Hospital admission (n, %)	19/26 (73.1%)	209/259(80.7%)	0.355
ICU admission (n, %) (*N* = 257)	1/21 (7.8%)	14/236 (5.1%)	0.826
Non-invasive mechanical ventilation (n. %)(*N* = 220)	5/15 (33.3%)	69/205 (33.7%)	0.979
Endotracheal intubation (n, %) (*N* = 254)	1/21 (4.8%)	9/233 (3.9%)	0.839
Resolution and discharged (*N* = 264)	23/24 (95.8%)	146/240 (60.8%)	<0.001
Death	1/26 (3.8%)	80/259 (30.9%)	<0.001
Hydroxychloroquine (*N* = 238)	15/21 (71.4%)	160/217 (73.7%)	0.819
Lopinavir/ Ritonavir(*N* = 239)	6/21 (28.6%)	86/218 (39.4 %)	0.328
Steroids(*N* = 284)	5/26 (19.2%)	86/258 (33.3%)	0.142
Azithromycin(*N* = 239)	7/21 (33.3%)	86/218 (39.4%)	0.583
Tocilizumab(*N* = 239)	1/21 (4.8%)	19/218(8.7%)	0.532
Beta-Interferon(*N* = 239)	1/21 (4.8%)	11/218 (5.0%)	0.955
Remdesevir(*N* = 239)	0/21 (0%)	1/218 (0.5%)	0.756

*Resolutio*n* = discharged home at the time of reporting. Expressed as percentages, mean ± SD or median [IQR p25-75].SD: standard deviation; IQR: interquartile range; ACEIs: angiotensin converting enzyme inhibitors; ARBs; anti-AT1 receptor antagonists, NSAIDs: non-steroidal anti-inflammatory agents.
